# DNA Dynamics under Periodic Force Effects

**DOI:** 10.3390/ijms22157873

**Published:** 2021-07-23

**Authors:** Alexander Svidlov, Mikhail Drobotenko, Alexander Basov, Eugeny Gerasimenko, Vadim Malyshko, Anna Elkina, Mikhail Baryshev, Stepan Dzhimak

**Affiliations:** 1 Department of Radiophysics and Nanothechnology, Physics Faculty, Kuban State University, 350040 Krasnodar, Russia; svidlov@mail.ru (A.S.); mdrobotenko@mail.ru (M.D.); son_sunytch79@mail.ru (A.B.); 013194@mail.ru (A.E.); baryshev_mg@mail.ru (M.B.); 2 Laboratory of Problems of Stable Isotope Spreading in Living Systems, Federal Research Center the Southern Scientific Center of the Russian Academy of Sciences, 344006 Rostov-on-Don, Russia; intro-2@rambler.ru; 3 Department of Fundamental and Clinical Biochemistry, Kuban State Medical University, 350063 Krasnodar, Russia; 4 Department of Technology of Fats, Cosmetics, Commodity Science, Processes and Devices Kuban State Technological University, 350072 Krasnodar, Russia; rosmaplus@gmail.com

**Keywords:** DNA, mathematical model, open states, rotational movements of nitrogenous bases, dynamics of a double-stranded DNA molecule

## Abstract

The sensitivity of DNA to electromagnetic radiation in different ranges differs depending on various factors. The aim of this study was to examine the molecular dynamics of DNA under the influence of external periodic influences with different frequencies. In the present paper, within the framework of a mechanical model without simplifications, we investigated the effect of various frequencies of external periodic action in the range from 10^11^ s^−1^ to 10^8^ s^−1^ on the dynamics of a DNA molecule. It was shown that under the influence of an external periodic force, a DNA molecule can perform oscillatory movements with a specific frequency characteristic of this molecule, which differs from the frequency of the external influence *ω*. It was found that the frequency of such specific vibrations of a DNA molecule depends on the sequence of nucleotides. Using the developed mathematical model describing the rotational motion of the nitrogenous bases around the sugar–phosphate chain, it is possible to calculate the frequency and amplitude of the oscillations of an individual DNA area. Such calculations can find application in the field of molecular nanotechnology.

## 1. Introduction

A DNA molecule is a complex structure that stores genetic information. It is known that the sensitivity of DNA to the effects of electromagnetic radiation in different ranges differs depending on the intensity of the transcription of the genes that form it and the phase of the cell cycle, especially in the S-phase, when DNA replication occurs [1,2,3]. For example, the effect of the microwave spectrum on bacterial DNA during the S-phase with a frequency resonant with the natural frequency of DNA torque [4] leads to the termination of replication and cell death, while when exposed to sub terahertz and terahertz ranges, for each of the nitrogenous bases, the absorption line corresponding to the resonance of their hydrogen bonds is revealed [5,6,7,8,9]. In turn, oscillations of DNA chains (one chain relative to another) can also correspond to low-frequency and mid-frequency radio bands, and after studying the data of the IR spectroscopy of a DNA molecule, it was shown that absorption lines are mainly associated with the vibrations of individual interatomic bonds in the molecule [10,11,12]. At the same time, the absorption of electromagnetic waves by purine and pyrimidine cycles in the UV spectrum (up to 400 nm) is due to quantum transitions between energy levels, which in general can change the activity of genes and regulate the rate of cell division [13].

Considering such a wide range of various factors influencing the molecular dynamics of DNA and causing periodic effects of different frequencies on it, the study of such effects based on the example of the gene encoding interferon alpha 17 seems to be relevant for understanding the principles of altering the oscillations of DNA with different nucleotide sequences at a changing frequency of external influence.

It should be noted that the experimental study of DNA dynamics is limited by the spatial resolution of the available biophysical instruments [14]. For this reason, the main research method is mathematical modelling [15]. Despite the large number of simplifications, mathematical modelling methods allow for taking into account various aspects of the behavior and functioning of the DNA molecule with great accuracy.

Mechanical models play an important role in the theoretical study of the internal dynamic of a DNA molecule. In this case, DNA bases are represented in the form of pendulums, and the interactions between the bases are described by replacing the hydrogen bonds with elastic bonds. Such an approach was proposed in [16], and further studies have allowed for developing a mechanical model for describing the dynamics of DNA, taking into account various forms of external influence, different effects of dissipation, and the inhomogeneity of the macromolecule itself [17].

In the work of [18], the results of the study of forced angular oscillations of nitrogenous bases under the influence of an external periodic force are presented. For a DNA molecule with averaged characteristics in the chains of nitrogenous bases, in the framework of the continuum approximation, solutions have been obtained, from which it could be deduced that the frequency of the angular deviations is equal to the frequency of an external periodic action.

In a previously published study [19], it was shown that simplifications of the mathematical model of DNA, such as averaging its characteristics, significantly affect the solution, including the frequency of the angular deviations.

In the present paper, within the framework of a mechanical model without simplifications [20,21], we investigated the effect of various frequencies of external periodic action on the dynamics of a DNA molecule.

## 2. Mathematical Model

To model the dynamics of a DNA molecule, we used a mathematical model that describes the rotational motion of nitrogenous bases around the sugar–phosphate chain. To build such a model, we used an analogy between a DNA molecule and a mechanical system consisting of two chains of interconnected pendulums. In this case, the rotating pendulums corresponded to nitrogenous bases, and the elastic thread to which these pendulums are attached corresponded to the sugar–phosphate chains of the DNA molecule; the hydrogen bonds of a pair of complementary nitrogenous bases corresponded to an elastic bond of the corresponding pair of pendulums [18].

This mathematical model includes the following Newton equations:

(1) $$I_{1}^{i}\frac{d^{2}\varphi_{1}^{i}\left(t\right)}{dt^{2}}=K_{1}^{i}\left[\varphi_{1}^{i-1}\left(t\right)-2\varphi_{1}^{i}\left(t\right)+\varphi_{1}^{i+1}\left(t\right)\right]-\;k_{12}^{i}R_{1}^{i}\left(R_{1}^{i}+R_{2}^{i}\right)sin\varphi_{1}^{i}-k_{12}^{i}R_{1}^{i}R_{2}^{i}sin\left(\varphi_{1}^{i}-\varphi_{2}^{i}\right)+\;F_{1}^{i}\left(t\right),\;i=\bar{2,n-1},$$

(2) $$I_{1}^{1}\frac{d^{2}\varphi_{1}^{1}\left(t\right)}{dt^{2}}=K_{1}^{1}\left[\varphi_{1}^{2}\left(t\right)-\varphi_{1}^{1}\left(t\right)\right]-\;k_{12}^{1}R_{1}^{1}\left(R_{1}^{1}+R_{2}^{1}\right)sin\varphi_{1}^{1}-k_{12}^{1}R_{1}^{1}R_{2}^{i}sin\left(\varphi_{1}^{1}-\varphi_{2}^{1}\right)+\;F_{1}^{1}\left(t\right),$$

(3) $$I_{1}^{n}\frac{d^{2}\varphi_{1}^{n}\left(t\right)}{dt^{2}}=K_{1}^{n}\left[\varphi_{1}^{n-1}\left(t\right)-\varphi_{1}^{n}\left(t\right)\right]-\;k_{12}^{n}R_{1}^{n}\left(R_{1}^{n}+R_{2}^{n}\right)sin\varphi_{1}^{n}-k_{12}^{n}R_{1}^{n}R_{2}^{n}sin\left(\varphi_{1}^{n}-\varphi_{2}^{n}\right)+\;F_{1}^{n}\left(t\right),$$

(4) $$I_{2}^{i}\frac{d^{2}\varphi_{2}^{i}\left(t\right)}{dt^{2}}=K_{2}^{i}\left[\varphi_{2}^{i-1}\left(t\right)-2\varphi_{2}^{i}\left(t\right)+\varphi_{2}^{i+1}\left(t\right)\right]+\;k_{12}^{i}R_{1}^{i}\left(R_{1}^{i}+R_{2}^{i}\right)sin\varphi_{2}^{i}-k_{12}^{i}R_{1}^{i}R_{2}^{i}sin\left(\varphi_{1}^{i}-\varphi_{2}^{i}\right)+\;F_{1}^{i}\left(t\right),\;i=\bar{2,n-1},$$

(5) $$I_{2}^{1}\frac{d^{2}\varphi_{2}^{1}\left(t\right)}{dt^{2}}=K_{2}^{1}\left[\varphi_{2}^{2}\left(t\right)-\varphi_{2}^{1}\left(t\right)\right]+\;k_{12}^{1}R_{2}^{1}\left(R_{1}^{1}+R_{2}^{1}\right)sin\varphi_{2}^{1}1-k_{12}^{1}R_{1}^{1}R_{2}^{1}sin\left(\varphi_{1}^{1}-\varphi_{2}^{1}\right)+\;F_{2}^{1}\left(t\right),$$

(6) $$I_{2}^{n}\frac{d^{2}\varphi_{2}^{n}\left(t\right)}{dt^{2}}=K_{2}^{n}\left[\varphi_{2}^{n-1}\left(t\right)-\varphi_{2}^{n}\left(t\right)\right]+\;k_{12}^{n}R_{2}^{n}\left(R_{1}^{n}+R_{2}^{n}\right)sin\varphi_{2}^{n}-k_{12}^{n}R_{1}^{n}R_{2}^{n}sin\left(\varphi_{2}^{n}-\varphi_{1}^{n}\right)+\;F_{2}^{n}\left(t\right).$$

Here,

$\varphi_{j}^{i}\left(t\right)$—angular deviation of the *i*-pendulum of the *j*-chain, counted counterclockwise, at time *t*;

$I_{j}^{i}$—moment of inertia of the *i*-pendulum of the *j*-chain;

$R_{j}^{i}$—distance from the center of mass of the *i*-pendulum of the *j*-chain to the thread;

$K_{j}^{i}$—constant characterizing the torque of the *i*-section of the *j*-thread;

$k_{12}^{i}$—constant characterizing the elastic properties of the connection of the *i*-pair of pendulums;

$F_{j}^{i}\left(t\right)$—external influence on the *i*-pendulum of the *j*-chain at time *t*.

n—the number of pairs of pendulums in the system under consideration.

In Equations (1)–(6), the first term to the right of the equal sign describes the force action from the elastic thread on the *i*-pendulum, and the second and third terms from the paired pendulum (the fourth term) are the external force action. The magnitude of the external influence is taken equal to $F_{j}^{i}\left(t\right)=-\beta_{j}^{i}\frac{d\varphi_{j}^{i}}{dt}\left(t\right)+F_{0}cos\omega t$, where the term $-\beta_{j}^{i}\frac{d\varphi_{j}^{i}}{dt}\left(t\right)$ models the effects of dissipation caused by the interaction with the liquid surrounding the DNA molecule, and the term $F_{0}cos\omega t$ models external periodic influence.

Note that the proposed model does not provide for the emergence of open states due to the breaking of hydrogen bonds.

We will add the initial conditions to Equations (1)–(6):

(7) $$\varphi_{1}^{i}\left(0\right)=\varphi_{1,0}^{i},\;\frac{{d\varphi }_{1}^{i}}{dt}\left(0\right)=\varphi_{1,1}^{i},$$

(8) $$\varphi_{2}^{i}\left(0\right)=\varphi_{2,0}^{i},\;\frac{{d\varphi }_{2}^{i}}{dt}\left(0\right)=\varphi_{2,1}^{i},\;i=\bar{1,n}.$$

For definiteness, we will assume that at *t* = 0 the system is in equilibrium, that is, in the initial conditions (7) and (8)

$$\varphi_{1,0}^{i}=\varphi_{1,1}^{i}=\varphi_{2,1}^{i}=0,\;\varphi_{2,0}^{i}=\pi ,\;i=\bar{1,n}.$$

Problems (1)–(8) are the Cauchy problems for a system of 2n ordinary differential equations. 

## 3. Influence of Periodic Exposure on DNA Dynamics

We investigated the influence of the frequency of the external periodic influence *ω* on DNA dynamics using the example of the gene encoding interferon alpha 17. For this gene n = 980, the base sequence of the first chain and the values of the coefficients of Equations (1)–(6) (Table 1) were taken from the work of [18], and the amplitude of the external periodic impact was F_0_ = 0.5 × 10^−22^ J.

In the same article, Equations (1)–(6) are replaced by their continual analogs, which, after averaging the coefficients of the resulting equations and simplifying the pattern of the connection between the bases, allows for obtaining angular deviations in the following form:

(9) $${\bar{\varphi }}_{i}\left(t\right)=A_{i}\cos\left(\omega t+\varphi_{0i}\right),\;i=1,\;2$$

where $A_{i}$ and $\varphi_{0i}$ are some constants. Thus, in [18], the frequency of oscillation of the angular deviations of the base chains ${\bar{\varphi }}_{1}$ and ${\bar{\varphi }}_{2}$ was equal to the frequency of the external influence ω.

In the present paper, we studied the effect of the frequency of the external periodic action *ω* on the DNA dynamics, without simplifying assumptions based on the numerical solution of Problems (1)–(8) obtained by the Runge–Kutta method of the fourth order (using a computer program with the certificate of state registration no. 2019667043). 

**Figure 1:**
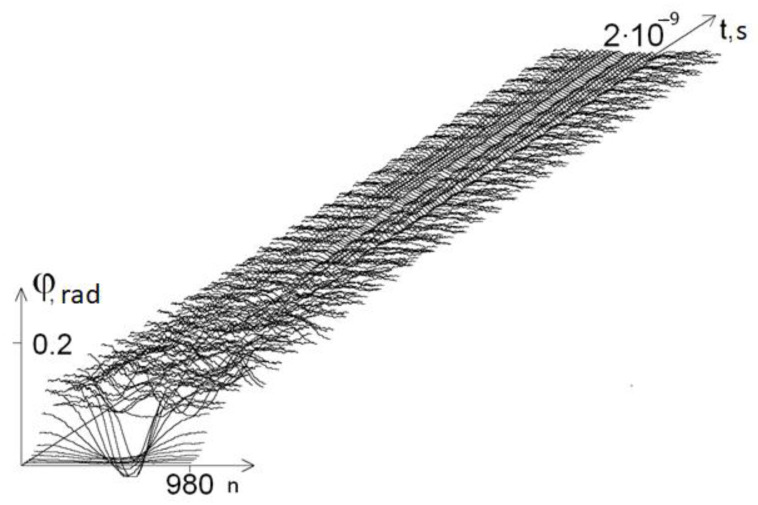
Graph of the angular deviations of the first chain of the gene encoding interferon alpha 17 at *ω* = 1 × 10^−11^ s^−1^.

Figure 1 shows a graph of the angular deviations of the first chain of the DNA molecule at *ω* = 1 × 10^−11^ s^−1^.

The dependence of the solutions to Problems (1)–(3) (or the dynamics of the DNA molecule) on the frequency of the external influence *ω* were determined using the average angular deviations of the first chain of the DNA molecule, as follows.

**Figure 2:**
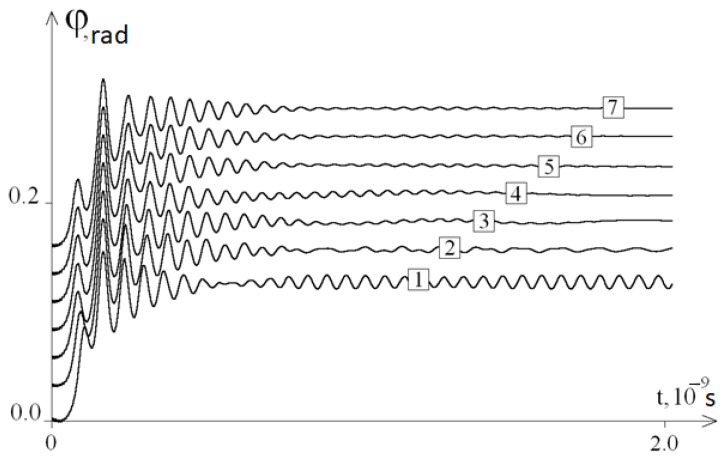
The average angular deviations (with vertical shift) of the first chain of the DNA molecule for different values of the frequency of external periodic impact: 1—*ω* = 10^11^ s^−1^, 2—*ω* = 5·10^10^ s^−1^, 3—*ω* = 10^10^ s^−1^, 4—*ω* = 5·10^9^ s^−1^, 5—*ω* = 10^9^ s^−1^, 6—*ω* = 10^8^ s^−1^, and 7—*ω* = 0.

Figure 2 shows the graphs of the average angular deviations of the first chain for different values of frequency ω; it can be seen that these graphs are not described by Equalities (4). 

At 0 < t ≤ 0.5 × 10^−9^ s, all graphs were similar to each other, and at t ≥ 0.5 × 10^−9^ s, the graphs contained periodic components of different frequencies. At ω ≥1 × 10^10^ s^−1^ (graphs 3–6), the amplitudes of the periodic components decreased with increasing t, and the frequencies depended on the time t and a little on ω. Note also that these frequencies are close to the frequency of the periodic component of graph 7 of the average angular deviations under a constant force action (ω = 0).

Note that the frequency of the periodic component of the oscillations of the DNA molecule was not constant and changed over time. A quantitative analysis of the dependence of this frequency on time was not considered in this work.

**Figure 3:**
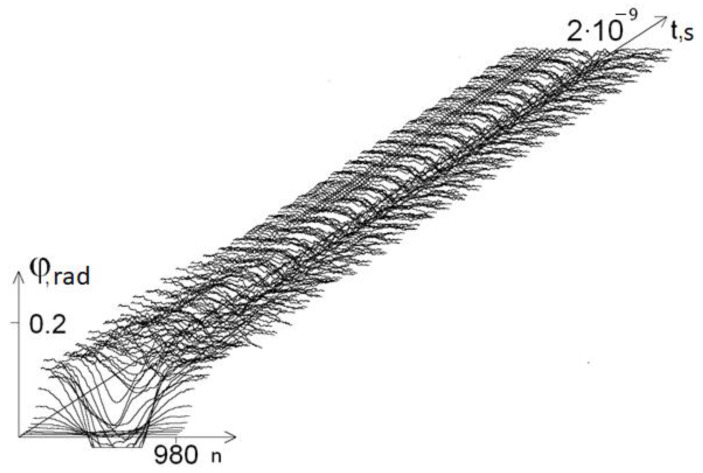
Graph of the angular deviations of the first DNA chain with 40A substitution at *ω* = 10^11^ s^−1^.

**Figure 4:**
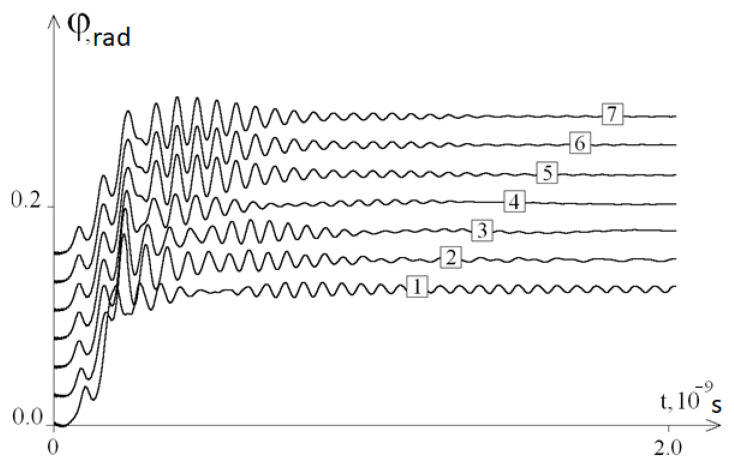
The average angular deviations (with a vertical shift) of the first DNA chain with 40A substitution for different orders of p frequency of external periodic exposures: 1—*ω* = 10^11^ s^−1^, 2—*ω* = 5 × 10^10^ s^−1^, 3—*ω* = 10^10^ s^−1^, 4—*ω* = 5·10^9^ s^−1^, 5—*ω* = 10^9^ s^−1^, 6—*ω* = 10^8^ s^−1^, and 7—*ω* = 0.

We conducted a qualitative analysis of the dependence of the oscillation frequency on the composition of the DNA molecule. To perform this, we changed the initial DNA molecule, replacing 40 bases in the middle of the first chain of the molecule with A (adenine) bases. Figure 3 shows a graph of the angular deviations of such a molecule at *ω =* 10^11^ s^−1^, and Figure 4 shows the graphs of the average angular deviations of the first chain at different values of *ω*. 

**Figure 5:**
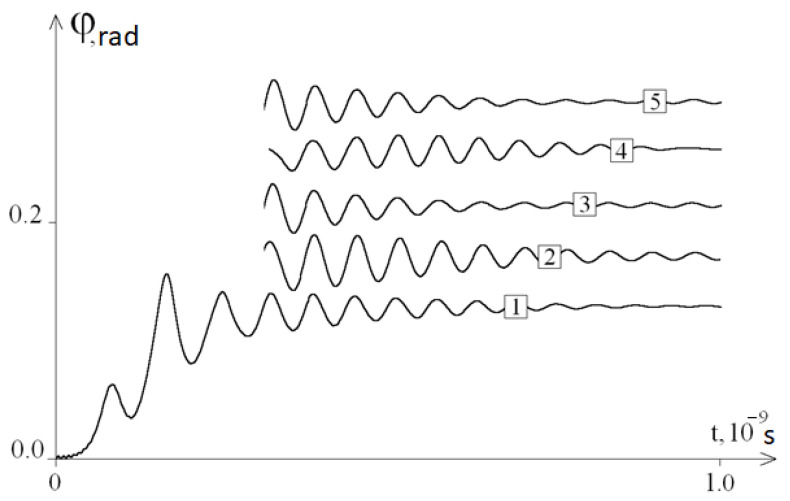
The average angular deviations (with a vertical shift) of the first DNA chain at *ω =* 10^8^ s^−1^ of the initial DNA (1) and fragments of the graphs for DNA with replacement with (2) 40A, (3) 40T, (4) 40G, and (5) 40C.

**Figure 6:**
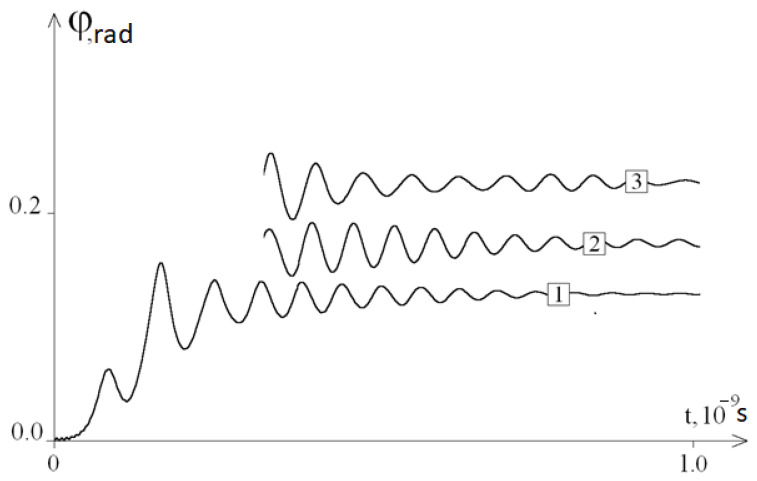
The average angular deviations (with a vertical shift) of the first DNA chain at *ω =* 10^8^ s^−1^ of the initial DNA (1) and fragments of the graphs for DNA with replacement of (2) 40A and (3) 100A.

Figure 5 shows the graphs fragments of the average angular deviations of the first chain at *ω =* 10^8^ s^−1^ for molecules in which 40 bases in the middle of the initial molecule were replaced by bases A, T, G, and C, and Figure 6, which shows molecules where 40 and 100 bases in the middle of the initial molecule were replaced by bases A. It can be seen from these figures that the frequency and amplitude of the oscillations of the DNA molecule depended on the nucleotide sequence.

## 4. Discussion

So, based on the results of the study, it can be concluded that the developed mathematical model fully confirms the existence of the phenomenon, as a result of which, after removing the system from the equilibrium position, periodic fluctuations of the average angular deviations of the DNA chain are observed. The frequency of these angular deviations is independent from ω (Figure 2). Thus, modelling of the molecular dynamics indicates a pattern describing the frequency of specific oscillations of a DNA molecule under periodic external influence. The study of the features of these specific DNA oscillations by modelling with its different molecular composition (different nucleotide sequences) revealed the presence of various patterns (Figure 4) that are different from the initial fluctuations in the amplitude of the mean angular deviations of DNA, which was due to the replacement of 40 nitrogenous bases to adenine in the middle of its molecule (from the 470th to 510th nucleotides). This phenomenon of molecular dynamics demonstrates the dependence of specific DNA oscillations primarily on the nucleotide sequence, but not on the frequency of the periodic external influence *ω*. 

The obtained results indicate a number of features of the influence of periodic external force effects on the molecular dynamics of DNA, including taking into account the change in the sensitivity of this molecule in the case of the formation of certain specific sequences of different lengths of homologous nitrogenous bases (A, T, C, and G).

First of all, the data presented in the work demonstrate the dependence of the frequency and amplitude of oscillations of a DNA molecule both on the specific nucleotide sequence (for example, homologous areas of each type of nitrogenous bases, Figure 5) and on different lengths of homologous sequences (Figure 6). At the same time, Figure 5 shows that the value of the average angular deviations of the first chain increases in the DNA molecule, which has a substitution of nitrogenous bases in the middle (from the 470th to the 510th nucleotide) for areas with the length of 40 nucleotides in the following row: 40C > 40G > 40T > 40A. In addition, they all exceed the mean angular deviations of the initial DNA molecule, which has a heterogeneous sequence of nitrogenous bases. In general, these data indicate both a lower (by 24% or more) sensitivity of heterogeneous DNA sequences to external periodic force effects, and a greater vulnerability of areas with nitrogenous bases of three hydrogen bonds (cytosine and guanine) in comparison with the sequences formed from adenine and thymine, the average angular deviations of which were 1.2–1.9 times lower than that of the molecules with the replacement of 40 consecutive nitrogenous bases exclusively by cytosine and guanine, each containing three hydrogen bonds (Figure 5).

An analysis of the data of the angular deviations of DNA at the same intensity of periodic external influence at *ω =* 1 × 10^8^ s^−1^, obtained by replacing 100 nitrogenous bases with adenine in the middle of the DNA molecule (from 440th to 540th nucleotides), showed that there is an increased aptitude for the average angular deviations of the first DNA chain by 1.3 times compared with the replacement of 40 nitrogenous bases with adenine in the same DNA molecule (Figure 6). The revealed feature indicates that the formation of longer sequences from homologous nitrogenous bases in the DNA structure leads to an increase of its average angular deviations under external periodic influences, the intensity of which is in the range *ω* = 1 × 10^8^ s^−1^. 

Such an aptitude increase in angular deviations can increase the rate of formation of open states and bubbles in DNA, and can be one of the reasons for an increase in the vulnerability of complementary nitrogenous bases as a result of a weakening of their hydrogen bonds in the area through the appearance of bubbles and open states under the influence of additional unfavorable external factors (prooxidants, chemical mutagens, radiation, etc.). All of the above-mentioned may be due to the fact that repulsive forces can arise between the strands of the double DNA of the helix, observed, for example, in the field of external electromagnetic waves. Moreover, at certain resonance frequencies, the repulsive forces acting on the strands of the double helix lead to the damage and rupture of the strands of the molecule, or in the case of their longitudinal propagation (action of external forces along the axis of the DNA helix), stretching and unwinding of the double helix may occur [13,22,23], which makes nitrogenous bases much more vulnerable to damage under the influence of various mutagenic factors. Taking into account the fact that the natural frequency of the torque of a gene depends on the length of the DNA helix that forms it and, therefore, this value is specific for different types of cells and their genes, the calculation of the indicators of the natural frequency of DNA oscillations, for example, in the oncogenes or genes of microorganisms, followed by the effect of an external electromagnetic field of the corresponding intensity, can lead to the suppression of the expression of genes of bacteria and oncoproteins, i.e., to a decrease in the multiplication of biopathogens, and a decrease of the growth of tumor cells [24,25,26]. In addition, the determination of the equilibrium of a double helix under the impact of external influences is also of interest for calculating the optimal parameters of the structure of artificial magnetic substances and bianisotropic metamaterials [27,28]. In this connection, the created mathematical model is of interest for various fields of science (medicine, biochemistry, nanotechnology, etc. [29]).

## 5. Conclusions

Thus, in the present study, it was shown that under the influence of an external periodic force, a DNA molecule can perform oscillatory movements with a specific frequency characteristic of this molecule, which differs from the frequency of the external influence *ω*. It was also found that the amplitude of the mean angular deviations in the gene encoding interferon alpha 17 is at least 24% less than the analogous indicators observed when 40 nitrogenous bases are replaced by homologous ones in the middle of the area (from the 470th to the 510th nucleotides), and decreases in the following row: 40C > 40G > 40T > 40A. In addition, when modelling periodic external influences with a frequency in the range of ω ≤ 1 × 10^8^ s^−1^, it was demonstrated that for nucleotide sequences with 40 nitrogenous bases with three hydrogen bonds, the average amplitude of the angular deviations are 1.2–1.9 times more than in the areas formed from adenine or thymine. The study showed that replacing 100 nitrogenous bases with adenine in the middle of the gene encoding interferon alpha 17 (from the 440th to 540th nucleotides) increased the amplitude of the mean angular deviations by 1.3 times compared with the replacement of 40 nitrogenous bases with adenine in the middle of the same gene.

In addition, taking into account the sequence of nucleotides and the length of the gene, using the developed mathematical model describing the rotational motion of nitrogenous bases around the sugar–phosphate chain, it is possible to calculate the frequency and amplitude of the oscillations of an individual DNA area to determine its resonance frequency. Such calculations can be applied in the field of molecular nanotechnology, for example, to calculate the resonance frequency of a molecule when modifying the initial nucleotide sequence, as well as to determine the resonance frequencies of foreign genes (oncogenes, genes of pathogenic microorganisms, etc.) in biological objects in order to be used later to suppress resonant electromagnetic radiation, which inhibits transcription and replication in foreign cells [30].

## Figures and Tables

**Table 1:** Equation coefficients (1)–(6).

Type of Base	A	T	G	C
$I\cdot 10^{-44},\;\mathrm{kg}\cdot m^{2}$	7.61	4.86	8.22	4.11
R, Å	5.80	4.80	5.70	4.70
$K\cdot 10^{-18}$ , J	2.35	1.61	2.27	1.54
$k_{12}^{H}\cdot 10^{-2},\;N/m$	6.20	6.20	9.60	9.60
$\beta \cdot 10^{-34},\;J\cdot s$	4.25	2.91	4.10	2.79
